# Crystallographic snapshots of ligand binding to hexameric purine nucleoside phosphorylase and kinetic studies give insight into the mechanism of catalysis

**DOI:** 10.1038/s41598-018-33723-1

**Published:** 2018-10-18

**Authors:** Zoran Štefanić, Marta Narczyk, Goran Mikleušević, Saša Kazazić, Agnieszka Bzowska, Marija Luić

**Affiliations:** 1 Division of Physical Chemistry, Ruđer Bošković Institute, Bijenička 54, Zagreb, 10000 Croatia; 2 Division of Biophysics, Institute of Experimental Physics, Faculty of Physics, University of Warsaw, Pasteura 5, Warsaw, 02-093 Poland

## Abstract

Purine nucleoside phosphorylase (PNP) catalyses the cleavage of the glycosidic bond of purine nucleosides using phosphate instead of water as a second substrate. PNP from *Escherichia coli* is a homohexamer, build as a trimer of dimers, and each subunit can be in two conformations, open or closed. This conformational change is induced by the presence of phosphate substrate, and very likely a required step for the catalysis. Closing one active site strongly affects the others, by a yet unclear mechanism and order of events. Kinetic and ligand binding studies show strong negative cooperativity between subunits. Here, for the first time, we managed to monitor the sequence of nucleoside binding to individual subunits in the crystal structures of the wild-type enzyme, showing that first the closed sites, not the open ones, are occupied by the nucleoside. However, two mutations within the active site, Asp204Ala/Arg217Ala, are enough not only to significantly reduce the effectiveness of the enzyme, but also reverse the sequence of the nucleoside binding. In the mutant the open sites, neighbours in a dimer of those in the closed conformation, are occupied as first. This demonstrates how important for the effective catalysis of *Escherichia coli* PNP is proper subunit cooperation.

## Introduction

Purine nucleoside phosphorylase (PNP, purine nucleoside orthophosphate ribosyl transferase, EC 2.4.2.1) has a crucial role in the purine salvage pathway^1^. It catalyses the reversible phosphorolytic cleavage of the glycosidic bond of purine (2′-deoxy) nucleosides, generating the corresponding free base and (2′-deoxy) ribose 1-phosphate^1,2^. The biologically active form of this enzyme, with only one known exception - PNP from *Thermus thermophilus* HB27^3^, is always oligomeric: homotrimers are characteristic mostly for mammals, while homohexamers are typical for most bacteria. The arrangement of subunits in the structure of a PNP hexamer is such that two of them donate two amino acids (His4 and Arg43 in *E. coli*) to each other, thus completing the active site of its neighbour, effectively forming a dimer which possesses an approximate 2-fold symmetry. Three such dimers are then arranged by a 3-fold symmetry axis to form a hexamer.

The molecular mechanism by which this enzyme accomplishes its biological function is particularly intricate. In addition to the phosphorolysis being a two-substrate and two-product reversible reaction, with communication between monomers forming a dimer^1^, the mechanism is further complicated by the higher order communication, *i.e*. an allosteric cross-talk between dimers forming the hexamer^4,5^. The way in which this allosteric communication is achieved is still unclear, but the complexity is reflected in an ever increasing number of different arrangements of active site conformations between monomers in the available crystal structures of hexameric PNPs^6^.

**Figure 1:**
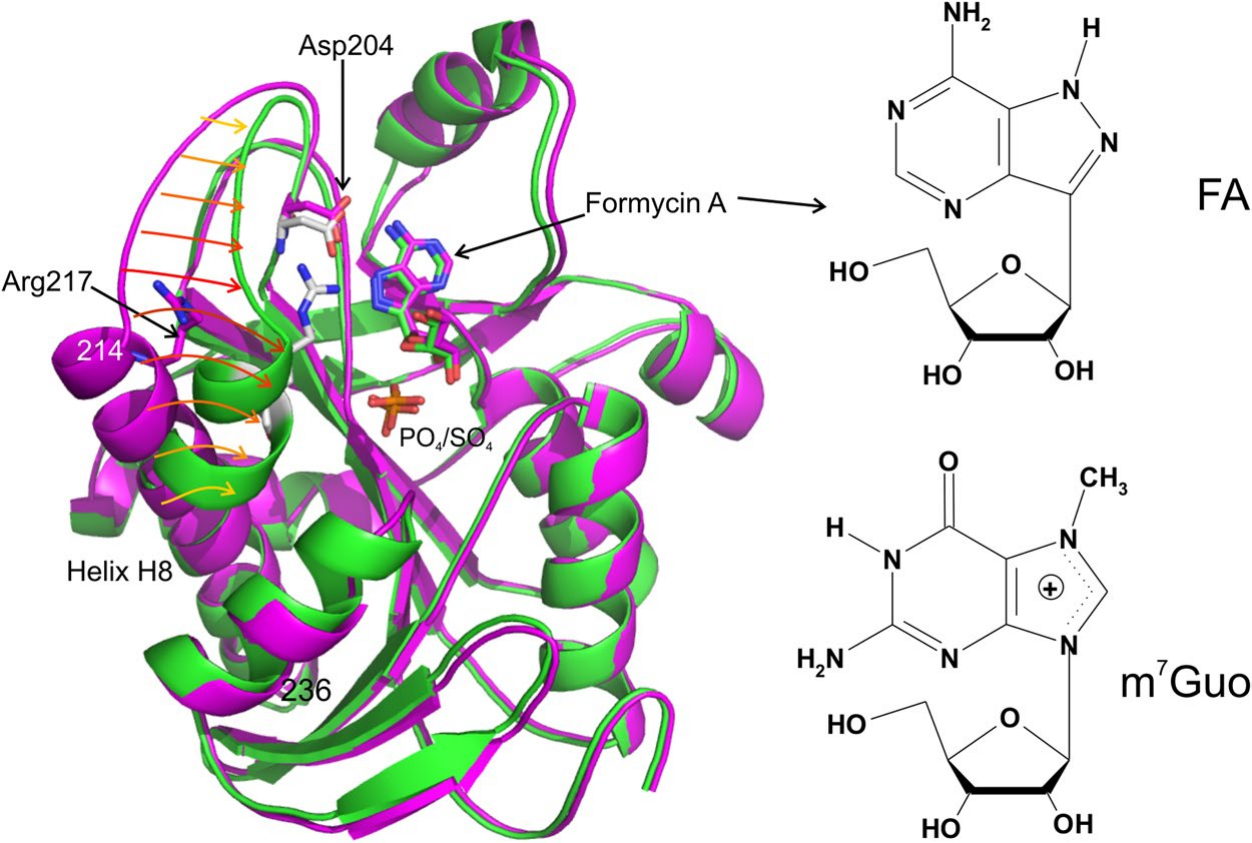
Left panel: Open and closed conformation of *E. coli* PNP. Monomers (**A**) (green) and (**B**) (magenta) of the wild-type *E. coli* ternary complex with phosphate/sulphate and FA structure, WT-6(P/S)-6FA, are overlapped to show the difference between closed and open active site conformation. The positions of catalytic amino acids Asp204 and Arg217 mutated to Ala in the double mutant structures are shown. The extent of movement of the loop and segmentation of the helix H8 is indicated by arrows. Right panel: structure of PNP ligands used in this study, PNP inhibitor FA, the N9 deaza analogue of the natural enzyme substrate adenosine, and 7-methylguanosine, the substrate bearing the positive charge on the five-membered ring of purine as the result of methylation at position N7.

The catalytic mechanism of PNP, especially that of *E. coli* PNP, has been in the focus of our study for many years^2,5,7–16^. It has been firmly established^5,14^ that the initial step in the catalysis involves breaking a single α-helix H8 (214–236) into two segments (Fig. 1). One of the segments (residues 214–219) moves in the direction of the binding pocket, helped by the formation of a γ-turn by the residues 220–222, thus partially closing the active site. The remaining part of the H8 α-helix stays in place (residues 223–236). The movement of the segmented part of the H8 α-helix is essential as it brings the side chain of the conserved amino acid Arg217 within a hydrogen bonding distance to the also conserved Asp204 residue as the initial step in the catalysis^5^. This triggers the proton transfer from Asp204 side-chain, which is initially in the acidic form, to the purine base at the N7 position. The crystal structure of the *E. coli* enzyme, which served for the formulation of the above mechanism^5^, was the first to capture the coexistence of the two conformations of the active site, open and closed, in the hexameric PNPs.

The active site in closed conformation was supposed to bind more strongly both PNP substrates, phosphate and nucleoside, than the active site in open conformation. It was proposed that the initial binding and proper alignment of the substrates occurs in the open conformation, while catalytic action requires the conformation to be closed^5^. Three different methods were used to validate this model: site-directed mutagenesis, kinetic studies, and X-ray crystallography^14^. These experiments supported earlier predictions that the binding of phosphate and its interaction with Arg24 are necessary for the conformational change to occur. Namely, the crystal structure of the binary complex of Arg24Ala mutant with the phosphate showed that all six active sites remained in the open conformation, indicating that the binding of phosphate in the absence of Arg24 is not sufficient for a conformational change to take place.

Solution studies have shown that *E. coli* PNP exhibits the negative cooperativity in binding of substrates and their analogues: for example phosphate^17^ and two non-cleavable analogues of adenosine, formycin A (FA, see Fig. 1, right) and its methylated form. Formycins are strong inhibitors of hexameric PNPs^5,10,18^. Although stoichiometry of stronger and weaker binding sites, as well as weaker binding constants, were not unequivocally determined for them, it was shown without any doubt that only part of the sites bind phosphate, FA and their analogues, with micromolar dissociation constants and makes the binding to the remaining active sites less likely^5,10,14,17,18^. However, in crystal structures of ternary complexes reported up to now^5,13,19^, it has never been observed that only part of the active sites were occupied by nucleosides.

Here we present a study regarding the wild type enzyme and another active site mutant, double mutant Asp204Ala/Arg217Ala (DM), showing how markedly these two mutations can change the binding and catalytic properties of PNP. As already mentioned, amino acids Asp204 and Arg217 are responsible for the protonation of the nucleoside substrate. Mutation of one or both of these residues markedly lowered the activity of PNP *vs*. natural substrates^14^, showing that they cooperate mutually in the protonation of the purine base, in agreement with the mechanism we proposed previously^5^.

Detailed study of kinetics of DM *versus* natural substrate, adenosine, and guanosine analogue, which bears a methyl group in the N7 position, introducing a positive charge on the five-membered ring of purine (Fig. 1) and therefore not requiring protonation, revealed an unexpected behaviour. Namely, the kinetics with phosphate (P_i_) as the variable substrate, in case of both proteins, is best described with the same model, allosteric with negative cooperativity. But in the case of catalysis with nucleoside as the variable substrate, we observe not only different kinetic constants for both enzyme forms, but also the model describing the catalysis changes from the Michaelis-Menten for WT to allosteric with probably positive cooperation for DM mutant. In this work we present these findings, and solution and crystallographic studies that explain why these two enzyme forms exhibit such striking differences.

## Results and Discussion

### Kinetic properties of WT and DM

**Figure 2:**
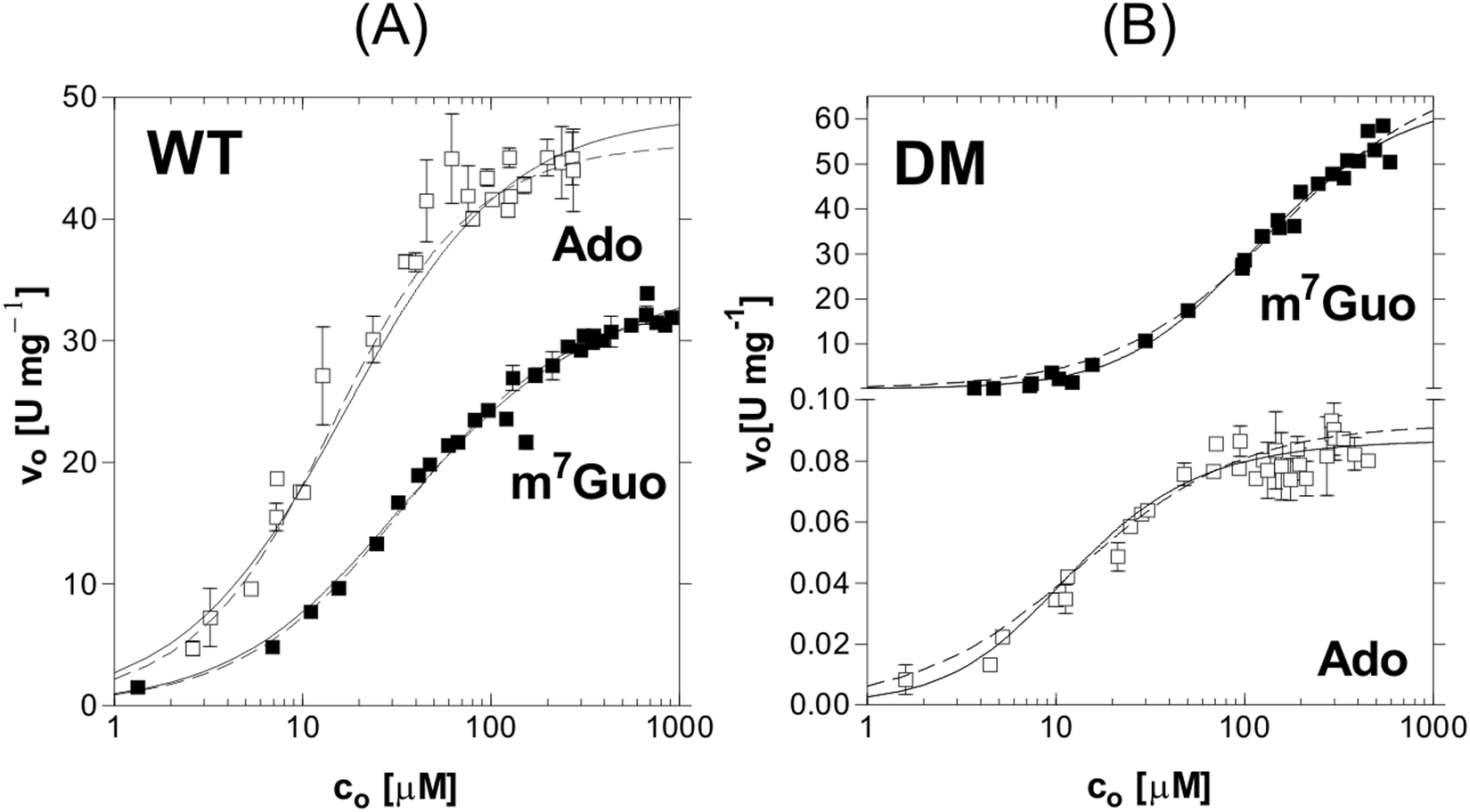
Activity of *E. coli* PNP and its DM mutant, with nucleoside, Ado (□) and m^7^Guo (■), as variable substrates, and a constant, saturating concentration of the second substrate, phosphate. The reactions were conducted at 25 °C in 50 mM Hepes buffer at pH 7.0 and followed by direct spectrophotometric assays. Fitting was performed with the equation (1), representing allosteric interaction between binding sites (solid lines) and with the Michaelis-Menten (MM) equation (dashed lines). For WT enzyme (**A**), MM equation described the data properly, while for DM (**B**) and data sets shown here F-test indicated that equation (1) was more appropriate (see also Materials and Methods). Error bars represent a standard deviation of the average rate from several independent measurements. For the DM and m^7^Guo as variable substrate averaging of rates lead to high errors of fitted parameters therefore fitting was performed separately for each experiment and one such experiment is depicted on this figure. Kinetic parameters obtained from the fitting of MM and (1) equations, for WT and DM, respectively, are shown in Table 1.

Catalytic and kinetic properties of the wild-type (WT) and the Asp204Ala/Arg217Ala double mutant (DM) were studied with adenosine (Ado), which is a natural substrate, and 7-methylguanosine (m^7^Guo), which has a methyl group in the N7 position of the purine that is protonated by the enzyme, according to the mechanism proposed^5^ and verified by studying the active-site mutants^14^. As a result of methylation, m^7^Guo bears a positive charge on the purine ring (Fig. 1), hence its protonation by the enzyme is not necessary. The kinetic data obtained are summarized in Table 1 and depicted in Fig. 2.

**Table 1:** Kinetic parameters fitted to initial velocity data obtained at pH 7.0, 50 m*M* Hepes buffer, 25 °C for WT and DM mutant of *E. coli* PNP with (upper part) phosphate as the variable substrate (Ado at 0.22 m*M* or m7Guo 0.25 m*M*), and (lower part) nucleoside as the variable substrate (phosphate at 50 m*M*); using allosteric interaction site model (equations (1, 2)). Errors of the parameters determined in this study are fitting errors.

Enzyme	Nucleoside as constant substrate	K_m1_ [m*M*]	V_max1_ [U mg^−1^]	K_m2_ [m*M*]^b^	V_max2_ [U mg^−1^]	a	b
**Phosphate as variable substrate**							
Non-recombinant^a^	m^7^Guo	0.015 ± 0.002	16 ± 1	0.135 ± 0.045	11 ± 1	9	0.69
WT	m^7^Guo	0.015 ± 0.004	10.8 ± 0.7	4.0 ± 3.7	8.2 ± 0.6	267 ± 232	0.76 ± 0.06
DM	m^7^Guo	0.066 ± 0.014	32.5 ± 2.0	6.8 ± 1.9	44.2 ± 2.3	103 ± 26	1.4 ± 0.1
WT	Ado	0.23 ± 0.10	20.8 ± 3.6	3.6 ± 2.6	19.3 ± 0.9	15.9 ± 8.2	0.93 ± 0.14
DM	Ado	0.28 ± 0.17	0.021 ± 0.006	15.2 ± 8.3	0.050 ± 0.009	55 ± 29	2.4 ± 0.5
**Enzyme**	**Phosphate as constant substrate**	**K**_**m1**_ **[m** ***M*** **]**	**V**_**max1**_ **[U mg** ^**−1**^ **]**	**K**_**m2**_ **[m** ***M*** **]** ^**b**^	**V**_**max2**_ **[U mg** ^**−1**^ **]**	**a**	**b**
**Nucleoside as variable substrate**							
Non-recombinant^b,c^	m^7^Guo	0.036 ± 0.006	30.2 ± 1.7	c	c	c	c
WT^c^	m^7^Guo	0.027 + 0.002	22.7 ± 0.3	c	c	c	c
DM^d^	m^7^Guo	~0.110^e^	~18^e^	0.024 ± 0.008	29.2 ± 1.5	0.22 ± 0.20	~1.5^e^
WT^c^	Ado	0.028 ± 0.004	51.1 ± 2.3	c	c	c	c
DM^d^	Ado	0.013 ± 0.009	0.011 ± 0.025	0.005 ± 0.007	0.043 ± 0.020	0.38 ± 0.24	∼4.0^e^

Parameters K_m1_ and V_max1_ are apparent Michaelis constant and apparent maximal velocity observed with one site occupied by the variable substrate, and with the neighbouring site free, while K_m2_ = aK_m1_ and V_max2_ = bV_max1_ characterise one site when the neighbouring site is occupied. In most cases, the kinetic parameters shown in the table are an average from several independent experiments.

^a^From Modrak-Wójcik *et al*.^20^.

^b^From Bzowska *et al*.^9^.

^c^Michaelis-Menten model was sufficient to describe the kinetic data in this case.

^d^The kinetic model for the nucleoside as variable substrate may be even more complicated, since for some data sets several minima with very similar sum of squares were found. The most important finding for the purpose of this report is that, in contrast to WT, the Michaelis-Menten model was not sufficient to describe the initial velocity kinetic data for DM and nucleoside as variable substrate.

^e^Errors are high, >50%, so a more precise value of the parameter could not be given.

Literature data report that *E. coli* PNP phosphorolysis studied with phosphate as the variable substrate does not proceed according to the Michaelis-Menten model^14,20^. It is rather described by the model of allosteric interaction between active sites as a result of binding phosphate (equations (1 and 2), Materials and methods), with both sites exhibiting similar maximal velocities, V_max1_ ~ V_max2_, but with the second site characterized by a higher Michaelis constant than the first one, K_m2_ > K_m1_, hence with negative cooperation between two active sites.

The same phenomenon, *i.e*. non-Michaelis-Menten kinetics, is observed now for the phosphorolysis of Ado and m^7^Guo catalysed by DM (Table 1), despite the fact that catalytic efficiency *vs*. these substrates of WT and DM enzymes are very different. DM is not able to protonate Ado, hence its catalytic activity (maximal activity of the enzyme in the allosteric model used is 2V_max2_) is about 400-fold lower than that of WT (0.05 U mg^−1^ and 19.3 U mg^−1^, respectively). By contrast, the activity of DM *vs*. m^7^Guo is 5 times higher than that of WT (44.2 U mg^−1^ as compared to 8.2 U mg^−1^). As mentioned above, m^7^Guo carries a methyl group at position N7 of purine base. Such methylation changes charge distribution over purine ring (See Fig. 1) making it imitate the charge distribution of protonated Ado, Ino or Guo, a natural substrates, which normally, under pH around 7 are in neutral form. Hence, this particular substrate does not require protonation by the enzyme, and it seems logical, that mutation of two amino acids responsible for protonation, Asp204 and Arg217 into Ala, has even beneficial effect on the catalysis of m^7^Guo.

Because non-Michaelis-Menten kinetics, is observed for the phosphorolysis of Ado and m^7^Guo catalysed by both enzyme forms, we may conclude that the mechanism of interaction with phosphate is the same for WT and DM, in agreement with the fact that only nucleoside-binding site amino acids were mutated in DM.

The situation is completely different when we consider catalysis with nucleoside as the variable substrate (Fig. 2 and Table 1). When phosphate is at a constant and saturating concentration, the catalysis conducted by the WT enzyme (Fig. 2A) is sufficiently well described by the classical Michaelis-Menten equation. This suggests that in such optimal conditions the interaction between subunits is effective and very well synchronized. Catalysis occurs only in the closed sites, but phosphate is bound also to the neighbouring open site of the same dimer. In this way, the presence of two kinds of sites is not reflected in the kinetics of the reaction.

This is not the case with DM (Fig. 2B), for which Michaelis-Menten model is not sufficient to describe the kinetic data when nucleoside is the variable substrate, and the interacting sites model seems to be more appropriate. However, as opposed to the kinetics when phosphate is the variable substrate, in this case there is an indication that K_m2_ might be lower than K_m1_ (however errors are too big to unequivocally show it, see Table 1). It means that the site, which is occupied at a higher nucleoside concentration, might have a lower Michaelis constant, suggesting a possible positive cooperation between active sites of DM.

These findings prompted us to conduct further studies aimed at understanding how these two mutations affect the mechanism of catalysis and/or binding of ligands.

### Differential analysis of amide hydrogen/deuterium exchange for WT and DM

**Figure 3:**
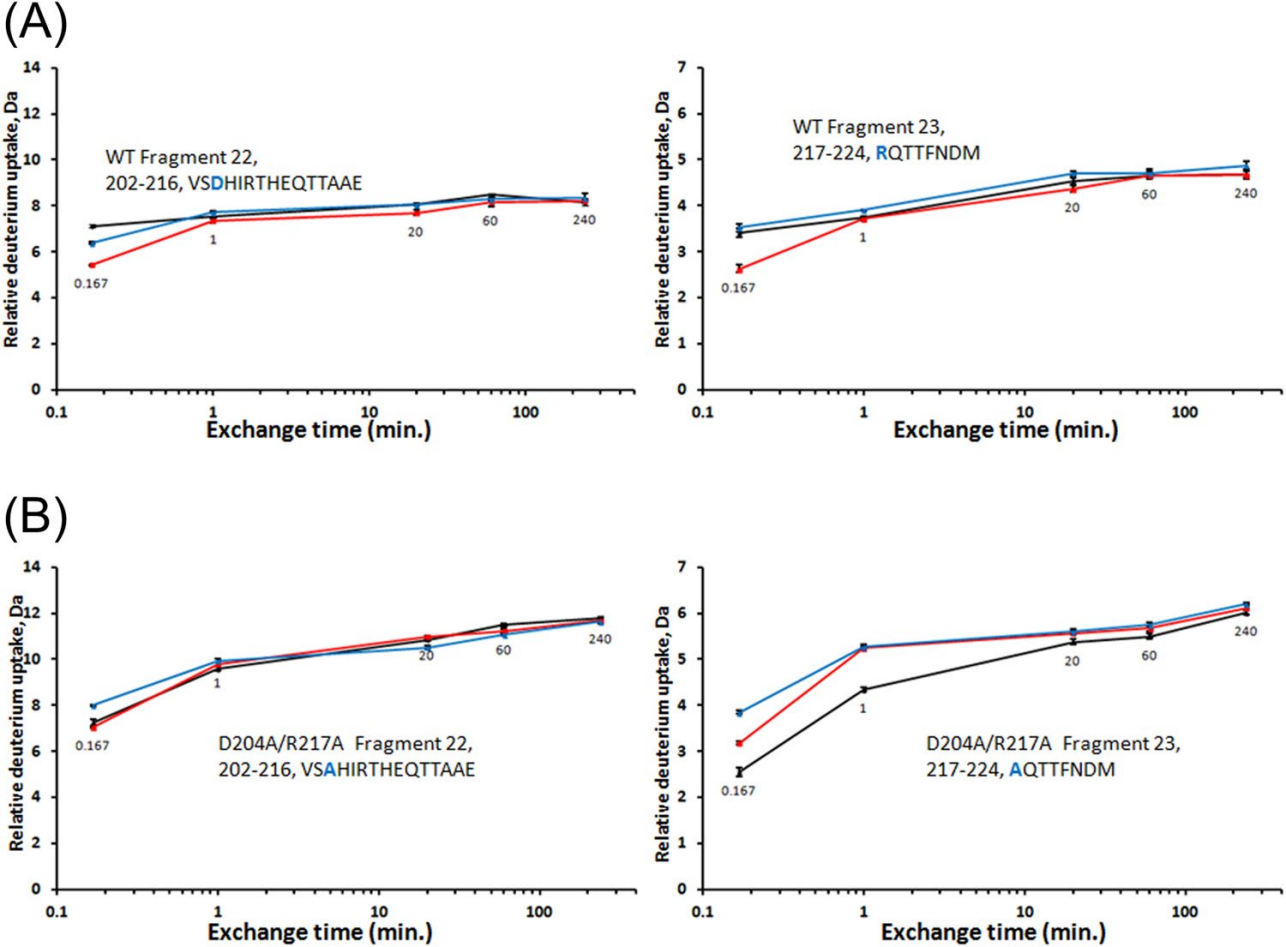
Deuterium uptake curve for unliganded protein (black trace), phosphate complex (red trace) and complex with phosphate and FA (blue trace). Each deuterium incorporation point is an average of 3 independent experiments. Analysis of the standard deviation for all triplicates provided a 98% confidence interval of about ±0.3 Da. Error bars were marked in the figure but are too small to be clearly visible. Deuterium incorporation graphs for (**A**) peptides 202–216 VSDHIRTHEQTTAAE and 217–224 RQTTFNDM of the wild-type *E. coli* PNP and (**B**) peptides 202–216 VSAHIRTHEQTTAAE and 217–224 AQTTFNDM of the *E. coli* PNP Asp204Ala/Arg217Ala mutant.

As shown previously^19^ for *E. coli* PNP, in the WT enzyme hydrogen/deuterium exchange rate is changing markedly upon the binding of ligands, and mutants can behave differently than the WT. A comparison of hydrogen/deuterium exchange data collected for WT and DM PNPs allowed us to detect differences in the flexibility of these two enzymes in the apo form, in binary complexes with phosphate (P_i_), and in ternary complexes with phosphate, and the non-cleavable analogue of adenosine, formycin A (FA, see Fig. 1, right), which, as mentioned in the Introduction, is a strong inhibitor of hexameric PNPs^5,10,18^. A difference in deuterium uptake between the three states of two protein forms (Fig. 3) is found just in two peptide fragments, p22 and p23, both holding a mutation site (Asp204Ala and Arg217Ala) and covering sequence regions of the WT *E. coli* PNP ^202^VSDHIRTHEQTTAAE^216^ for p22 and ^217^RQTTFNDM^224^ for p23. In all other fragments, changes induced by phosphate and FA binding are the same for both enzyme forms, WT^4^ and DM. Namely, phosphate binding induces a significant reduction in the conformational dynamics of the binary complex in those structure regions that contain amino acid residues establishing strong H-bonds with P_i_ in the active site. Phosphate forms strong hydrogen bonds with Arg24, Arg87, Arg43 (the last one form neighbouring monomer of the same dimer) and Ser90. In the closed active site of the WT, Arg217 and Asp204 are H-bonded between each other, while in the open conformation P_i_ makes H-bonds with Ser203 (p22) and - in WT only - with Arg217 (p23), that do not exist in the closed conformation. Exchange kinetics of peptides 22 and 23 is fast, and a dynamics slowdown upon P_i_ binding is visible only during the shortest time period of 10 seconds.

Differences detected in structure flexibility between WT and DM double mutant can be seen in fragments 22 (Fig. 3A, left) and 23 (Fig. 3A, right) because both peptides contain alanine (A) residues instead of charged aspartic acid (Asp, peptide 22) and charged arginine (Arg, peptide 23). Relative to the unliganded state (Fig. 3B, black line, right), at 10 s, the double mutant shows a higher deuterium uptake upon P_i_ binding for fragment 23 (Fig. 3B, red line, right). This is opposite to a decrease in deuterium uptake (Fig. 3A, red line, right) observed for WT fragment 23 at the same exchange time period. Due to the absence of Arg217, in DM the P_i_ charge cannot be neutralized as effectively as in the WT form, thus allowing water molecules to enter into the active site cavity and increase deuterium uptake relative to the unliganded form, as shown in Fig. 3. Structure region of the double mutant covered by peptide 22 is placed out of the active site cavity so that P_i_ charge solvation (Fig. 3B, red line, left) does not change deuterium uptake in this region relative to the unliganded form (Fig. 3B, black line, left).

After FA binding, the ribose part of the molecule establishes H-bonds with the side chain of Glu181, fixing motion of that part of the protein structure and causing a lower deuterium uptake for peptide 19 in both enzyme forms, WT^4^ and DM (not shown).

Differences in the conformational flexibility of WT and DM induced by FA binding can be observed again, like in the case of enzyme/P_i_ binary complex, for p22 and p23. All graphs show an increased deuterium uptake upon FA binding to the active site (blue line over red line) because hydrogen bonding rearrangement caused by the binding of this bulky molecule relaxes the overall structure rigidity obtained after entering P_i_ into the active site. Deuterium uptake for the ternary complex of the WT remains lower for p22 (Fig. 3A, blue line, left) or the same for p23 (Fig. 3A, blue line, right) as the uptake values for the unliganded form (Fig. 3A, black lines, left and right), but it is higher than the uptake measured for both peptides in the binary complex with phosphate (Fig. 3A, red lines, left and right). For the double mutant, deuterium uptake values are always higher for the ternary complex (Fig. 3B, blue lines, left and right) relative to either the unliganded form (Fig. 3B, black lines, left and right) or the binary complex with phosphate (Fig. 3B, red lines, left and right), indicating that the conformational flexibility arrest caused by P_i_ binding is not that pronounced in the double mutant as it is in the wild-type hexamer.

### Overall protein structures

**Figure 4:**
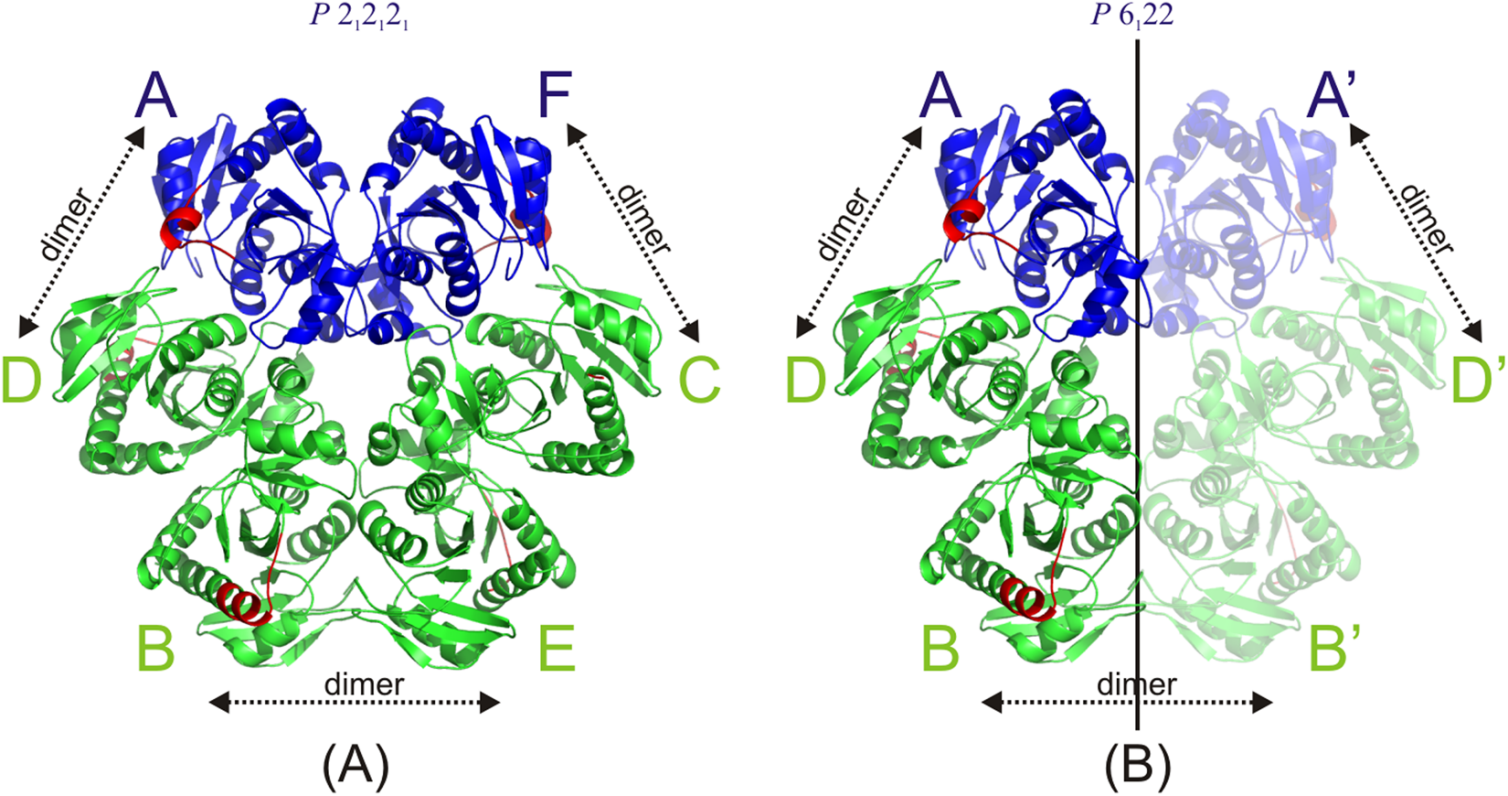
Schematic drawing of the hexamers of PNP in the two space groups. The colours indicate open (green) and closed (blue) conformations of monomers’ active sites. The amino acids of helix H8 (214–219) that move during the change of active sites’ conformation are shown in red. Arrows show which chains join together to form a dimer. (**A**) In the case of orthorhombic structures WT-6(P/S)-2FA, DM-6(P/S)-2FA and DM-6(P/S)-4FA, chains A and F are in closed conformation and chains B, C, D and E are in open conformation. (**B**) In the case of hexagonal structures WT-6(P/S)-6FA and DM-6(P/S)-6FA, only one half of the hexamer is crystallographically independent and the other half is generated by crystallographic two-fold axes (indicated by the paler half). In terms of open and closed conformations, in structures WT-6(P/S)-6FA and DM-6(P/S)-6FA, chain A (and by symmetry also A’) is in closed conformation, while B and D (and their symmetry equivalents B’ and D’) are in open conformation.

We have then conducted crystallographic studies to check whether a lack of two charged amino acids in the *E. coli* PNP active site can influence the conformations of the terminal α-helix, namely whether it can hamper segmenting this helix and closing active sites and whether this was an explanation of the above-described kinetic and hydrogen/deuterium exchange experiments. As a nucleoside, the same as in solution studies, FA, a non-cleavable structural analogue of the natural substrate adenosine was used (Fig. 1). It turned out that the conformation of both proteins with all the active sites occupied by both ligands was the same, with a closed conformation in one of three active sites (Fig. 4). Both ligands are in the same orientation in the active sites of WT and DM, and the conformation of crucial amino acids is very similar. This suggests that the differences introduced by these two mutations within the active sites and cooperation of the active sites within a hexamer are not assessable by the standard X-ray crystallographic experiments in which we are able to see the final effect of the ligand binding process, but not how the process proceeds.

We have made attempts to crystallise PNP in such a way to obtain structures with not all active sites filled with a nucleoside FA. The first attempt was to crystallise the protein with P_i_ only and to soak crystals for a short period of time in FA solution at various and decreasing concentrations of the nucleoside. Each time the structures obtained by this method contained FA molecules in each active site, though not always at full occupancy. Despite many trials and various FA concentrations in the soaking solutions, these results were the same.

Therefore, we tried another approach: setting crystals of ternary complexes, prepared at different FA to PNP/P_i_ complex concentration ratios. As a result of such co-crystallisation experiment, structures with part-of-the-sites occupancy of FA were finally obtained.

All PNP structures determined in this study show a homohexamer consisting of six chains of identical sequences of 238 amino acids (Fig. 4). They can be considered as trimers of dimers possessing approximately 32 point group symmetry. In the case of orthorhombic structures of the wild-type enzyme with two formycin molecules (WT-6(P/S)-2FA) and of the double mutant complexes with two and four formycin molecules per hexamer bound, (DM-6(P/S)-2FA and DM-6(P/S)-4FA, respectively) all six monomers are crystallographically independent, whereas in the case of hexagonal structures of the wild-type and the mutant with six formycin molecules bound by each hexamer (WT-6(P/S)-6FA and DM-6(P/S)-6FA) two halves of the hexamer are related by the crystallographic two-fold axis. As already observed for WT/P_i_ binary complex^14^, and WT ternary complexes with phosphate (sulphate) and FA^15^, and also FA derivatives^5^, each monomer can be found in either open or closed active site conformation (Fig. 1). In the active site pocket, phosphate and nucleoside binding sites can be distinguished. All phosphate binding sites of here described ternary complexes, both wild-type and double mutant, are occupied by phosphate or sulphate (precipitating agent in crystallisation). The collected experimental data for all five presented crystal structures do not allow a distinction between phosphate and sulphate ions, but in view of all the previous results^14,17^, phosphate ions are present in active sites in the closed conformation.

### Occupancy of the FA ligands

**Figure 5:**
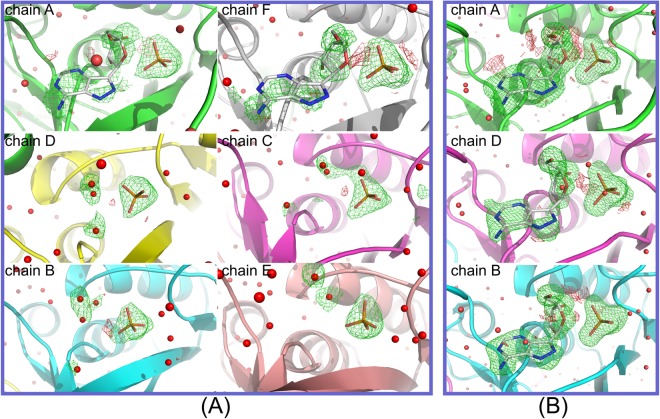
Electron density maps of the active site of the wild-type structures (**A**) WT-6(P/S)-2FA and (**B**) WT-6(P/S)-6FA given in approximately equal orientations. Maps shown are omit maps where the water and FA molecules in the active sites were omitted from the refinement (*i.e*. their occupancy was set to 0 and their coordinates were fixed). Maximum likelihood *mF*_o_*-DF*_c_ difference maps contoured at levels 3σ and -3σ are given in green and red, respectively. (**A**) In the case of structure WT-6(P/S)-2FA, chains A and F are in closed conformation and the presence of difference electron density indicates the presence of FA molecules in the active site. The remaining four chains (**B–E**) are in open conformation, and no continuous density could be identified where FA molecules could be placed. (**B**) Higher concentration of FA in the structure WT-6(P/S)-6FA results in filling the active sites of all the chains. Here also only chains A and F are in closed conformations. Difference electron density around FA molecule in chain D is slightly less noticeable than in the other open site B, probably indicating a lower binding affinity.

**Figure 6:**
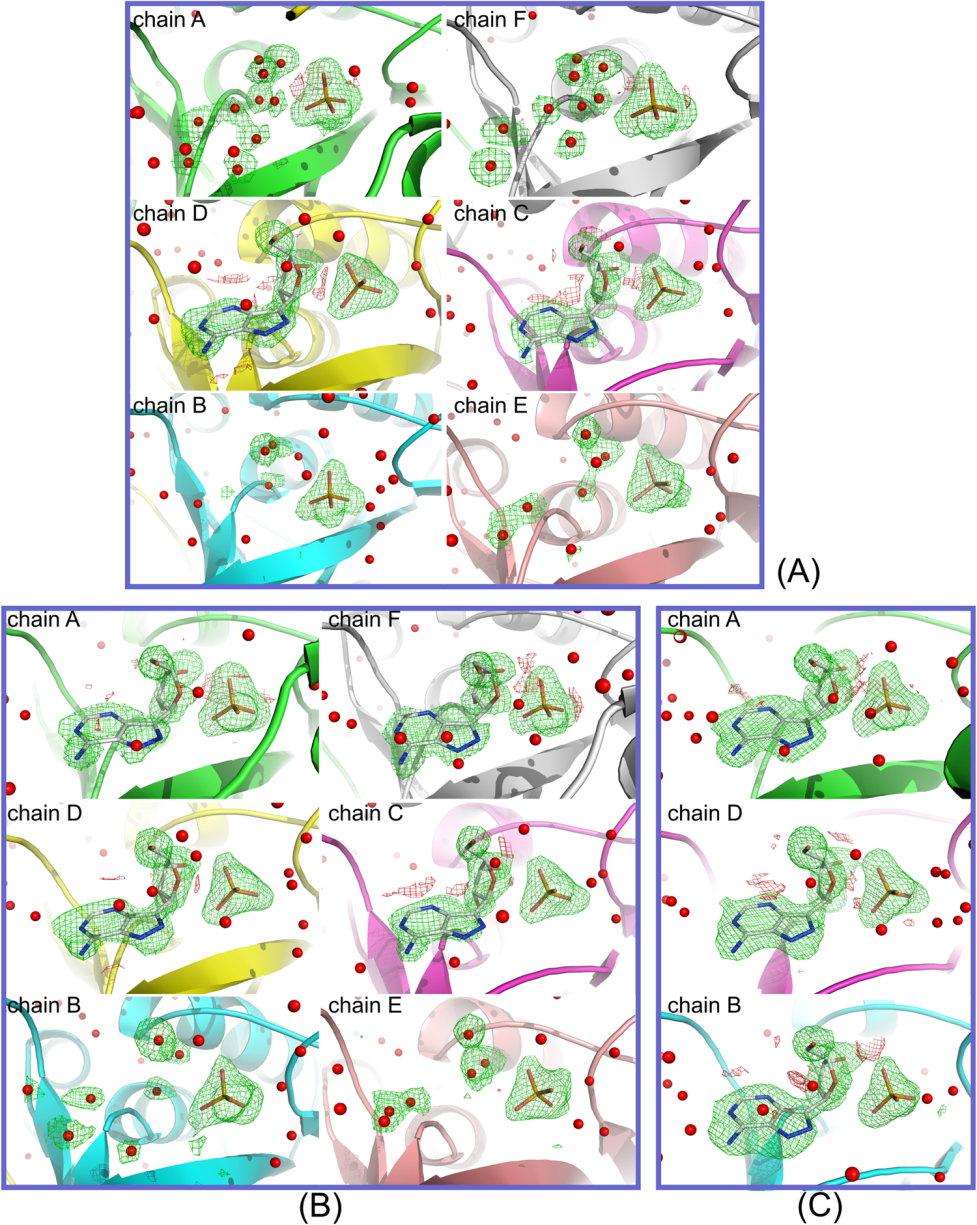
Electron density maps of the active sites of the double mutant crystal structures DM-6(P/S)-2FA, DM-6(P/S)-4FA and DM-6(P/S)-6FA given in approximately equal orientations. Maps shown are omit maps where the water and FA molecules in the active sites were omitted from the refinement (*i.e*. their occupancy was set to 0 and their coordinates were fixed). Maximum likelihood *mFo-DF*_*c*_ difference maps contoured at levels 3σ and -3σ are given in green and red, respectively. (**A**) In the structure DM-6(P/S)-2FA, the FA electron density is most pronounced in chains C and D and it clearly shows the presence of FA ligands. This is in marked contrast to chains A and F, which are in the usual closed conformation, but with electron density in the form of separate blobs, which are modelled as water molecules. Electron density is poorest in the open-open dimer B-E and is modelled as separate water molecules. (**B**) In the structure DM-6(P/S)-4FA, electron density around FA molecules is still most prominent in the open sites C and D, but this time also shows the presence of FA molecules in the corresponding closed chains A and F. Open-open dimer B-E remains occupied by water molecules only. (**C**) The concentration of FA molecules in the structure DM-6(P/S)-6FA is such to allow all active sites to be occupied and the electron density is clearly present in all six active sites of the hexamer (the other half of the molecule is symmetrically equivalent).

Difference electron density maps of the two wild-type structures are shown in Fig. 5, while those of the three double-mutant structures are shown in Fig. 6. Maps are omit maps calculated without water and FA molecules in the active site. Maximum likelihood 2*mF*_o_*-DF*_c_ maps are contoured at level 1σ, while *mF*_o_*-DF*_c_ difference maps are contoured at levels 3σ and -3σ. Estimating the occupancy of ligands in the active sites of the proteins only by crystallographic measurements is problematic at best^21,22^, therefore special attention was paid not to bias the analysis (see Materials and methods)^21,22^.

All of the structures have two monomers in closed conformations (A and F) next to each other but belonging to two different dimers, and four monomers in open conformations (B, C, D and E). In the orthorhombic structures, dimers A-D and F-C are closed-open dimers and B-E form an open-open dimer, while in the hexagonal forms A-D is the closed-open dimer and B-B’ is the open-open dimer formed by crystallographic two-fold symmetry (Fig. 4).

In the structure of WT-6(P/S)-2FA, the FA molecules are found only in the closed active sites A and F with final occupancies of 0.7 (Fig. 5A, Table 2). All of the active sites in the open conformation in this complex are occupied only by water molecules. This is the first time that the presence of nucleoside type substrate only in the active sites in closed conformations was shown. This strongly indicates a higher affinity of these sites for nucleosides in comparison with active sites in the open conformation and confirms our conclusion based on solution studies^5^, namely that negative cooperativity is also present in the binding of the nucleoside substrate.

**Table 2:** Location of FA molecules in the closed and open active sites of the *E. coli* PNP wild-type and double mutant hexamers (trimer of dimers), occupancy factors of FA molecules in the individual active sites (See also Figs 5 and 6), and data collection and refinement statistics.

	WT-6(P/S)−2FA	WT-6(P/S)-6FA	DM-6(P/S)-2FA	DM-6(P/S)-4FA	DM-6(P/S)-6FA
**Location of FA molecules and occupancy factors**					
Molar ration PNP:FA	1:0.14	1:0.57	1:0.4	1:0.6	1:1.2
Space group	*P* 2_1_ 2_1_ 2_1_	*P* 6_1_ 2 2	*P* 2_1_ 2_1_ 2_1_	*P* 2_1 _2_1 _2_1_	*P* 6_1 _2 2
Monomers in the asymmetric unit	A, B, C, D, E, F	A, D, B	A, B, C, D, E, F	A, B, C, D, E, F	A, D, B
Dimers	A-D; F-C; B-E	A-D; A’-D’; B-B’	A-D; F-C; B-E	A-D; F-C; B-E	A-D; A’-D’; B-B’
Occupancy factor in: A,F - closed sites	0.7; 0.7	0.8	no FA	0.6; 0.6	0.7
Occupancy factor in: D,C - open sites in the closed-open dimer	no FA	0.5	0.7; 0.7	0.6; 0.6	0.5
Occupancy factor in: B,E or B,B’ - open sites in the open-open dimer	no FA	0.4	no FA	no FA	0.6

The crystal structure of complex WT-6(P/S)-6FA was obtained with a higher concentration of FA compared to structure WT-6(P/S)-2FA, and this resulted in filling all the active sites, including those in the open conformation (Fig. 5B, Table 2). Difference electron density for omitted FA is best in the closed active site A, then in the open active site B, and finally somewhat poorer in the open active site D, which forms an open-closed dimer with site A. This is backed up by the values of occupancies of FA molecules, which in these three active sites are given as A:B:D = 0.8:0.5:0.4. This suggests that in the wild-type enzyme the active site B from the open-open B-B’ dimer exhibits a slightly higher affinity towards FA than the active site D that belongs to the closed-open A-D dimer (Fig. 5B).

The occupancies of FA molecules show a significantly different behaviour in the structures of the double mutant. Although the active sites A and F are closed in the structure of DM-6(P/S)-2FA, these sites are not first occupied by FA molecules. The only active sites that are occupied with FA molecules in structure DM-6(P/S)-2FA are open active sites D and C (Fig. 6A) from the closed-open dimer. Occupancy factor for both FA molecules in these active sites is 0.7. Closed active sites A and F and open active sites B and E are filled with water molecules only. The order of filling the active sites has changed with respect to the wild-type enzyme. It is evident that changing the two most important amino acids from the nucleoside binding site, Asp204 and Arg217, completely changed the affinity and probably cooperativity of the subunits (which is reflected in the kinetic properties, as discussed above). This demonstrates how only a few mutations of catalytically important amino acids can change the whole molecular mechanism of enzyme action.

When we used a higher concentration of FA molecules in the preparation of the ternary complex of the mutated protein, structure DM-6(P/S)-4FA, the FA molecules entered the closed active sites (Table 2). Here the occupancies of FA molecules are the same in closed sites A and F and in D and C, namely 0.6, and a visual inspection of difference electron density justifies this (Fig. 5B). The active sites of open-open dimer B-E are without FA molecules; instead the active site binds water molecules. This structure shows the second step in the filling of the active sites: after the open sites from the open-closed dimer, the closed sites from the same dimer show the highest affinity for FA.

Finally, with the highest concentrations of FA used in preparing the complexes, all the sites become occupied with FA molecules, as demonstrated by the electron density in structure DM-6(P/S)-6FA (Fig. 6C). Here all the sites clearly show the presence of the nucleoside and the refined occupancies of FA molecules are 0.7, 0.5, and 0.6 for chains A, B, and D, respectively. This is the third step in the process of FA binding to the *E. coli* PNP double mutant, which we could clearly identify by the X-ray crystallography method.

### Insight into the mechanism of catalysis

*E. coli* PNP is an excellent molecular machine conducting efficient catalysis of purine nucleosides glycosidic bond cleavage. As described above it is a homohexamer, built as a trimer of dimers, with all its subunits having the active site in the open conformation in the apo enzyme form^23^. Monomers in each dimer mutually donate two amino acids (His4 and Arg43) to complete their active sites. Thus, dimers in principle have all the features necessary to work independently. However, as we have shown recently, a trimer-of-dimers architecture is necessary to stabilize the three-dimensional structure of dimers and preserve their ability to form a closed conformation of the active site^16^. This in turn enables proper interactions of the enzyme with the nucleoside, and its protonation at position N7 of the purine base, which is a prerequisite for an efficient catalysis^5^. Here we take a step forward in understanding the molecular mechanism of this complicated catalyst by presenting crystallographic snapshots of ligand binding to individual subunits of the hexamer, WT enzyme and its DM - with mutations of two crucial amino acids responsible for the protonation of the nucleoside. We show that the sequence of events derived from X-ray structure is different in these two enzyme forms, and these results correlate well with the catalytic and kinetic properties of WT and DM. In the case of WT, at low nucleoside concentrations, the nucleoside is bound mostly in sites adopting a closed conformation, which facilitates protonation, hence also catalysis. In the case of DM, which is not able to protonate the substrate independently of active site conformation, as Asp204 residue is missing in this enzyme form (replaced by Ala), the nucleoside is bound mostly in active sites adopting the open conformation. However, m^7^Guo due to the methyl group at position N7 of the purine base, imitating protonation, already bears the positive charge. Therefore its further protonation by the enzyme, and the conformational change *i.e*. active site closing, enabling protonation, seems not just unnecessary, but are probably not even desirable, making additional, in this case not required, step in the catalytic cycle. And indeed, in the structure of the DM enzyme, active sites in the closed conformation are not the ones that are occupied first by nucleoside - open sites are those that bind nucleoside in the beginning. This change of nucleoside binding sequence by individual subunits makes DM an excellent catalyst, even better than the WT PNP, towards m^7^Guo. These results also show that residues Asp 204 and Arg 217 are responsible not only for protonation of the nucleoside but also influence the way how subunits of the enzyme cooperate with each other to conduct the efficient catalysis.

To the best of our knowledge, this is the first case reported in the literature where the sequence of ligand binding events at the active sites of the oligomeric enzyme (which also explains important catalytic properties of this molecule) was followed by crystallographic occupancy refinement of the series of enzyme-ligand complexes obtained by a step-wise slow increase of the ligand to protein concentration ratio in a co-crystallisation experiment, thus managing to fill two, four or all six active sites of the enzyme. This shows that a similar approach can also be used to follow ligand binding steps for other homooligomeric proteins showing negative cooperation between active sites, especially the so-called part-of-the-sites binding enzymes^24^.

## Materials and Methods

### Kinetic methods

All kinetic data were obtained as described previously^14^. Direct spectrophotometric method was used to follow phosphorolysis in 50 m*M* Hepes buffer pH 7.0, at 25°C of Ado (λ_obs_ = 265 nm, Δε  = −1690 M^−1^cm^−1^) and m^7^Guo (λ_obs_ = 260 nm, Δε  = −4600 M^−1^cm^−1^). Cuvettes with 1, 0.5, 0.2 and 0.1 cm path length were used to cover a broad nucleoside concentration range. Either the classical Michaelis-Menten model or the interacting site model was fitted (two equivalent forms used in literature are shown)^20,25^:

1 $${v}_{0}({c}_{0})=\frac{2\frac{{V}_{max1}{c}_{0}}{{K}_{m1}}+2\frac{b{V}_{max1}{c}_{0}^{2}}{a{K}_{m1}^{2}}}{1+2\frac{{c}_{0}}{{K}_{m1}}+\frac{{c}_{0}^{2}}{a{K}_{m1}^{2}}}$$

2 $${v}_{0}({c}_{0})=\frac{2{V}_{max1}{K}_{m2}{c}_{0}+2{V}_{max2}{c}_{0}^{2}}{{K}_{m1}{K}_{m2}+2{K}_{m2}{c}_{0}+{c}_{0}^{2}}$$

K_m1_ and V_max1_ are apparent Michaelis constant and apparent maximal velocity, respectively, observed when the neighbouring site is free; aK_m1_ =K_m2_ and bV_max1_ = V_max2_ characterise one site when both sites are occupied; c_0_ refers to the concentration of the variable substrate; and fitted parameters depend, in general, on the concentration of the co-substrate, which is held constant. The highest velocity observed at saturating concentration of the variable substrate is 2V_max2_.

Curve-fitting was carried out by the GraphPad Prism program (Intuitive Software for Science, San Diego, CA, USA). The more complicated model (Equations (1, 2)) was chosen if there was a statistically significant decrease in the sum of residuals as judged by the F-test at 95% confidence level. Phosphorolysis was measured several times for the same substrate concentration, and weights 1/SD^2^, where SD is a standard deviation of the average rate, were used in fitting. Since rates for high nucleoside concentration measured in thin cuvettes (0.2 and 0.1 cm) are prone to high error, in many cases weighting led to a different conclusion regarding the correct kinetic model when compared with the results of the cases in which no weights were used. With phosphate as the variable substrate, there was never any doubt that the interacting sites model described the data better, while with a nucleoside as the variable substrate, the result depends on the range of substrate concentration employed and on the weighting method used. For data sets shown in Fig. 1, for DM (right panel), F value is 14.2 and 8.2 for Ado and m^7^Guo, respectively. This indicates that there is only less than 0.0001% and 0.004% chance, respectively, that the data, that are described so much better by the interacting site model than by the Michaelis-Menten model, were obtained randomly. However, the kinetic model for of the nucleoside catalysis conducted by DM may be even more complicated than the one described by eqs (1, 2), since for some data sets several minima with very similar sum of squares were found. The most important finding for the purpose of this report is that, in contrast to WT, the Michaelis-Menten model was not sufficient to describe the initial velocity kinetic data for DM with nucleoside as the variable substrate (see Results and Discussion).

### Hydrogen/deuterium exchange mass spectrometry experiment

All HDX experiments were conducted at room temperature. H/D exchange reaction was initiated by diluting 5 µL of the protein stock solution (~75 mM protein concentration in 20 mM Tris-HCl, pH 7.4) in 45 µL of deuterated exchange buffer (20 mM Tris-HCl, pH 7.4 in D_2_O). The kinetics of the H/D exchange reaction was followed by measuring deuterium uptake at 5 time points: 10 s, 1 min, 20 min, 1 hour and 4 hours for peptides obtained from the digested protein. Deuterium exchange reactions were performed in triplicate, and the reported values for every peptide are an average of three independent reactions without correcting for back exchange. Statistical analysis was done according to Houde *et al*.^26^. Observed differences in deuterium incorporation at a particular time period which are greater than 0.3 Da are considered as significant. The reactions were quenched by lowering the pH_read_ to 2.5 and temperature close to 0 °C by adding 10 µL of pre-chilled stop buffer (2 M glycine buffer in D_2_O, pH 2.5) into the reaction solution. The established slow exchanging conditions (pH 2.5 and temperature ~ 0 °C) were maintained during 1.5 minutes of protein digestion performed using on-line Waters H/D exchange technology platform^27^ and a pepsin column (Poroszyme cartridge, 2.1 mm diameter and 30 mm length, Applied Biosystems) which was kept at room temperature. Trapping flow rate was 200 µL/min. Mass spectral information was collected throughout the peptic fragment elution profile from the reversed phase LC column (Acquity UPLC BEH C18 Column, 1.0 × 100 mm, 1.7 mm, Waters). At lowered temperature (~0 °C), the mobile phase composed of 93% of solvent A (0.1% formic acid in H_2_O) and 7% of solvent B (0.1% formic acid in acetonitrile) was pushed through the UPLC column at 55 µL flow rate while generating back pressure of close to 9000 psi. Gradient elution of the peptides was started at the solvent composition of A(93%)/B(7%), and the ratio was continuously changed during the following 7 minutes to achieve A(65%)/B(35%) composition. The ratio was rapidly changed during the next 50 seconds to reach A(15%)/B(85%) and was held at that level for the next 2.5 minutes. In the final chromatography step, during 50 seconds, solvent composition was reverted to A(93%)/B(7%) and retained until the end of the 12 minutes long elution. Mass spectra of the eluting peptides were acquired in the mass range of 300–1500 Da with continuous infusion of 0.5 µM leu-enkephalin solution for internal calibration. Undeuterated samples allowed for pepsin-digested peptides list to be assembled by using Waters ProteinLynx Global Server (Waters, Milford, MA). The amount of incorporated deuterium for each peptide was determined using Waters DynamX 2.0 software package. Differential comparisons of H/D exchange data collected for two protein forms (wild-type and Asp204Ala/Arg217Ala mutant of *E. coli* PNP), each in three states (unliganded, binary complex with P_i_, and ternary complex with P_i_ and FA), were carried out according to a procedure described elsewhere^28,29^.

### Preparation of ternary complexes for crystallisation

Recombinant wild-type and double mutant PNP proteins were prepared and purified as described previously^14^. Both proteins were concentrated to millimolar range (3–6 mM) in 10 mM citric buffer pH 7.0. Formycin A (5 mM or 10 mM) solution was prepared in the same buffer. As a source of phosphate, a 200 mM sodium phosphate buffer pH 7.0 was used. Ternary complexes of both WT and DM were obtained by mixing the appropriate volumes of protein, phosphate and FA solutions to reach a certain molar ratio of protein and FA. Phosphate concentration in all complexes was 5.5 times higher than protein concentration. The molar ratio between WT and FA in the prepared complexes WT-6(P/S)-2FA and WT-6(P/S)-6FA was 1:0.14 and 1:0.57, respectively. In previous studies where full ligand occupancy was observed, this ratio was 1:2.5^5^. For DM-6(P/S)-2FA, DM-6(P/S)-4FA, and DM-6(P/S)-6FA molar ratio was 1:0.4, 1:0.6 and 1:1.2, respectively. FA was added 10 minutes after adding phosphate buffer in all three DM complexes.

### Crystallisation and data collection

All complexes of PNP were crystallised at 18 °C using the hanging-drop vapour-diffusion method. All five complexes crystallised in the conditions with 50 m*M* citric buffer pH 5.2 and 32–34% ammonium sulphate (w/v). Crystals appeared after a month and were frozen in liquid nitrogen when they stopped growing. Prior to flash-freezing in liquid nitrogen, the crystals were soaked for a few seconds in a cryoprotecting solution containing 30% glycerol. In-house single-crystal data from complex WT-6(P/S)-2FA were collected on Xcalibur Nova R single-crystal diffractometer at 100 K using Cu Kα radiation (1.542 Å) and were processed and scaled using the CrysAlisPro software (Agilent Technologies). Diffraction data on complexes WT-6(P/S)-6FA and DM-6(P/S)-2FA were collected on BL14.1 operated by Helmholtz-Zentrum Berlin (HZB) at the BESSY II electron storage ring (Berlin-Adlershof, Germany) on a PILATUS 6 M detector using 2 s exposure and a single wavelength of 0.978 Å^30^. The data for complexes DM-6(P/S)-4FA and DM-6(P/S)-6FA were collected at ESRF BM14 beamline using MAR CCD 225 mm detector and a single wavelength of 0.954 Å. The data from synchrotron measurements were processed and scaled using the XDS package^31^. The crystals of proteins WT-6(P/S)-2FA, DM-6(P/S)-2FA, and DM-6(P/S)-4FA belonged to the orthorhombic space group *P* 2_1_ 2_1_ 2_1_ with one hexamer in the asymmetric unit, while the crystals of complexes WT-6(P/S)-6FA and DM-6(P/S)-6FA belonged to the hexagonal space group *P* 6_1_ 2 2 with one half of the hexamer in the asymmetric unit (Fig. 4). Full data collection, refinement and model statistics are given in Table 3.

**Table 3:** Data collection and refinement statistics.

	WT-6(P/S)-2FA	WT-6(P/S)-6FA	DM-6(P/S)-2FA	DM-6(P/S)-4FA	DM-6(P/S)-6FA
**Data collection**					
Space group	*P* 2_1_ 2_1_ 2_1_	*P* 6_1_ 2 2	*P* 2_1_ 2_1_ 2_1_	*P* 2_1_ 2_1_ 2_1_	*P* 6_1_ 2 2
Cell dimensions *a, b, c* (Å) *α*, β*, γ* (°)	61.9, 123.4, 189.0, 90, 90, 90	120.6, 120.6, 240.0, 90, 90, 120	62.7, 123.9, 188.7, 90, 90, 90	61.9, 124.2, 189.4, 90, 90, 90	120.7, 120.7, 239.5, 90, 90, 120
Resolution (Å)	28.6–2.3 (2.4–2.3)*	48.2–1.77 (1.8–1.7)	44.4–2.0 (2.1–2.0)	47.84–1.89 (1.96–1.89)	48.14–1.87 (1.94–1.87)
*R*_*meas*_	0.13 (0.31)	0.09 (0.46)	0.14 (0.48)	0.18 (0.80)	0.35 (0.81)
*R*_*pim*_	0.07 (0.23)	0.02 (0.11)	0.04 (0.16)	0.07 (0.64)	0.09 (0.23)
CC1/2	0.98 (0.74)	1.00 (0.97)	0.99 (0.94)	0.99 (0.38)	0.99 (0.73)
Mean *I*/*σ*(*I*)	8.7 (2.6)	42.2 (9.6)	16.6 (5.4)	10.9 (1.0)	8.2 (3.3)
Completeness (%)	99.6 (98.0)	97.3 (80.1)	99.5 (98.9)	98.8 (87.9)	99.8 (98.3)
Multiplicity	3.7 (2.3)	39.4 (32.7)	12.8 (13.1)	6.5 (3.6)	19.0 (20.5)
**Refinement**					
Resolution (Å)	28.6–2.3 (2.4–2.3)	48.2–1.77 (1.8–1.7)	44.4–2.0 (2.1–2.0)	47.8–1.9 (1.96–1.89)	48.1–1.9 (1.94–1.87)
No. reflections	241525 (14424)	3871879	1276422 (127853)	757641 (36393)	1617111 (170636)
*R*_*work*_/*R*_*free*_	0.18/0.24 (0.23/0.31)	0.16/0.18 (0.18/0.21)	0.15/0.20 (0.17/0.24)	0.17/0.21 (0.31/0.34)	0.16/0.19 (0.27/0.32)
Number of atoms	11783	6275	12135	12104	6447
Macromolecules	10910	5485	10716	10726	5512
ligands	68	72	98	131	87
water	805	718	1321	1247	845
Protein residues	1430	711	1414	1414	714
RMSD bonds (Å)	0.008	0.008	0.007	0.008	0.009
RMSD angles (°)	1.11	1.14	1.09	1.11	1.14

^*^Highest resolution shell is shown in parentheses.

Notation: WT-6(P/S)-2FA - ternary complex of wild-type protein with all 6 active sites in the homohexamer occupied by a phosphate/sulphate ion and 2 sites occupied by FA with similar connotations for other occupancies of FA in WT and DM ternary complexes.

### Structure determination and refinement

All of the crystal structures were determined and refined using the PHENIX suite^32^. The model for molecular replacement was PNP wild-type^5^ (PDB: 1K9S). No prior information was introduced as to whether the monomers were in closed or open active site conformation because the flexible part of the H8 α-helix was removed from the model. Special attention was paid in rebuilding the regions that had been excluded from the search model in order to clearly distinguish between open and closed active sites using Coot^33^. Figures of crystallographic structures were prepared in Pymol (The PyMOL Molecular Graphics System, Version 1.5.0.4 Schrödinger, LLC). Estimating the occupancy of ligands in the active sites of the proteins only by crystallographic measurements is problematic at best^21,22^. Because occupancy of a ligand is strongly correlated with the thermal displacement parameters of individual atoms, special attention was paid to taking this into account. In all the active sites, presence of PO_4_/SO_4_ molecules was clearly visible so their occupancy was set to 1.0 and their B-factors were refined. As the crystallisation conditions contained sulphate ions, and they are crystallographically indistinguishable from phosphate, we have used the formulas PO_4_/SO_4_ (P/S) to emphasize this. When difference electron density in the active site clearly evidenced the presence of FA molecules, they were placed into electron density with the occupancy less than 1.0, and the occupancy was refined while B-factors of the ligand atoms were fixed during occupancy refinement. The value at which B-factors of all the atoms were fixed was the average value for that ligand in the previous round of refinement. If electron density was not conclusive, both fitting the ligand and the water molecules was tried, and in the end the decision was made by a critical assessment of the fit. To estimate the uncertainty of the finally obtained occupancies, many refinement rounds with the starting occupancies of FA molecules set to initial random values between 0 and 1 were done. The final values of occupancies obtained in this way did not vary more than 0.01, irrespective of the starting occupancy value for all FA ligands in all the structures.

### Accession codes

The coordinates and structure factors have been deposited in the PDB (Protein Data Bank) with the following codes: 4TS3 for WT-6(P/S)-2FA, 4TS9 for WT-6(P/S)-6FA, 4TTA for DM-6(P/S)-2FA, 4TTI for DM-6(P/S)-4FA, and 4TTJ for DM-6(P/S)-6FA complex.
